# *Pasteurella multocida* Serotype D Infection Induces Activation of the IL-17 Signaling Pathway in Goat Lymphocytes

**DOI:** 10.3390/microorganisms12122618

**Published:** 2024-12-18

**Authors:** Yujing Fu, Yong Meng, Hejie Qian, Taoyu Chen, Xiangying Chen, Qiaoling Chen, Hongyan Gao, Churiga Man, Li Du, Si Chen, Fengyang Wang

**Affiliations:** Hainan Key Laboratory of Tropical Animal Reproduction & Breeding and Epidemic Disease Research, Animal Genetic Engineering Key Laboratory of Haikou, College of Tropical Agriculture and Forestry, Hainan University, Haikou 570228, China; 15383468682@163.com (Y.F.);

**Keywords:** *Pasteurella multocida* type D, peripheral blood lymphocytes, IL-17 signaling pathway, cytokines

## Abstract

(1) Background: Pasteurellosis is a global zoonotic bacterial disease, which has caused significant economic impacts in animal husbandry. Nevertheless, there is limited understanding of the immune response between goat peripheral blood lymphocytes (PBLs) and goat-derived *Pasteurella multocida* (*P. multocida*). (2) Methods: To investigate the immune response of host PBLs during infection with *P. multocida* type D, we established an *in vitro* cell model utilizing isolated primary goat PBLs. Utilizing this *in vitro* infection model, we employed an enzyme-linked immunosorbent assay (ELISA) to assess the cytokine profile variation in goat PBLs following infection. Meanwhile, RNA sequencing and quantitative PCR (qPCR) methods were employed to analyze the gene expression profile. (3) Results: The ELISA test results indicated that the expression levels of pro-inflammatory cytokines, such as IL-6, IFN-γ, CXCL10, and IL-17A, were significantly elevated within 12 h after infection with *P. multocida*. In contrast, the levels of the anti-inflammatory cytokine IL-10 were found to be reduced. RNA sequencing and functional enrichment analysis identified 2114 differentially expressed genes (DEGs) that were primarily associated with cytokine-cytokine receptor interactions, viral protein-cytokine interactions, and the IL-17 signaling pathway. Furthermore, protein-protein interaction (PPI) network analysis and qPCR highlighted *CD86*, *CCL5*, *CD8A*, *CXCL8*, *CTLA4*, *TNF*, *CD274*, *IL-10*, *IL-6*, *CXCL10*, *IFNG,* and *IL-17A* that were crucial for the response of PBLs to *P. multocida* infection. (4) Conclusions: This study systematically revealed the characteristics of PBLs in goats following infection with goat-derived *P. multocida* type D through the analysis of cytokines and gene expression, providing important theoretical insights for a deeper understanding of the defense mechanisms in goats against *P. multocida*.

## 1. Introduction

*Pasteurella* is ubiquitous in animal populations and is generally considered a component of the commensal microbiota found in the oral cavity, nasopharynx, and respiratory tract [1]. Worldwide, pasteurellosis is among the most common diseases that impact goats. Outbreaks typically result in high mortality rates among goats and lead to significant economic losses [2]. *Pasteurella* can cause both pneumonia-type pasteurellosis and systemic pasteurellosis in goats [3]. In Asia, the infection rates for sheep and goats rose from 4.2% and 6.7% in 2005 to 9.4% and 8.2% in 2019, respectively [4]. According to relevant reports, the mortality rate of pasteurellosis in lambs can be as high as 50% [5].

The pathogens responsible for caprine pasteurellosis are *P. multocida* and *M. haemolytica*, with *P. multocida* being the primary causative agent [6]. Under certain stress conditions, such as long-distance transportation, excessive fatigue, or improper feeding and management, respiratory viruses, mycoplasma, and other pathogens can trigger pasteurellosis. The infection mechanism of *P. multocida* is linked to various virulence factors, including capsular polysaccharides, lipopolysaccharides, outer membrane proteins, and trimer self-transporters [7]. *P. multocida* is classified into five serotypes, A, B, D, E, and F, based on its capsular antigen [8]. Goats are more susceptible to infection with subtypes A and D of *P. multocida* [9]. Our previous work demonstrated that, in a goat bronchial epithelial cell infection model, the *P. multocida* serotype D (Pm-HN01) strain caused a greater number of non-adherent cells and more pronounced morphological changes compared to the *P. multocida* serotype A (Pm-HN02) strain. In a mouse model, heart tissue from mice infected with *P. multocida* serotype D (Pm-HN01) exhibited more severe pathological damage than that from mice infected with *P. multocida* serotype A (Pm-HN02) [10,11].

Infectious diseases can lead to various forms of immune dysregulation in both innate and adaptive immunity. These dysregulations may include apoptosis-induced loss of immune effector cells, such as lymphocytes and dendritic cells, inactivation of monocytes, depletion of T cells, and an increase in myeloid-derived suppressor cells, as well as T regulatory cells [12]. Studies have demonstrated that 8 h after the inoculation of *P. multocida* strain P1062 (type A3) in goats, the count of peripheral blood lymphocytes was statistically significantly reduced [13]. This decrease in lymphocyte counts may result from the influx of these cells into tissues or bacterial products that induce apoptosis or cytolysis [14,15,16,17]. Our previous studies on the *P. multocida* serotypes D (Pm-HN01) found that one day after infection with *P. multocida* in goats, hematoxylin and eosin (HE) staining revealed a loss of structural characteristics in lung tissue, with the alveolar spaces filled with inflammatory exudates such as neutrophils and monocytes [18]. The study of hematological parameters in mice infected with *P. multocida* serotype A1 (P120) revealed significant changes in both the complete leukocyte count and the leukocyte differential count. Transient leukopenia was observed between 6 to 12 h post-infection, followed by progressive leukocytosis from 24 to 60 h. Lymphopenia was noted in the total leukocyte count. The percentage of apoptosis in circulating lymphocytes at various time points indicated that the apoptosis rate in the control group ranged from 0.8% to 1.4%. In contrast, the apoptosis rate in the infected group was measured at (18 ± 3.0%) at 24 h, (19.4 ± 3.6%) at 36 h, and (18.4 ± 3.1%) at 48 h, with a lower apoptosis rate observed in the later stages of infection [19]. This indicates a complex interaction between peripheral blood lymphocytes and *P. multocida*.

Researchers can utilize *in vitro* cell models to precisely control the conditions of cell culture, including temperature, pH, and nutrient levels. This controlled environment facilitates the study of cell behavior and responses under specific conditions. By employing high-throughput RNA sequencing of human peripheral blood mononuclear cells stimulated with *Mycobacterium tuberculosis* antigens (Mtb-Ag) alongside control samples, researchers analyzed the immune pathways related to Mtb-Ag stimulation [20]. Using T cell mitogens to activate peripheral blood mononuclear cells from Saanen goats and subsequently infecting them with various virulent strains of *Pestedes Petits Ruminants Virus*, researchers identified key immune factors associated with the *in vitro* virulence of PPRV through transcriptomic and proteomic analyses [21]. Research on the stimulation of CD4^+^ T lymphocytes isolated from peripheral blood by recombinant multi-killing *Pasteurella* toxin suggests that bacteria can exploit the high plasticity of T cell subtypes to enhance their disease-causing ability, potentially gaining advantages in survival and reproduction [22]. The study of RNA sequencing to investigate the effects of the chicken *reticuloendothelial virus* (REV) on the immune response and proliferation of lymphocytes from chicken peripheral blood *in vitro* has laid the foundation for uncovering the molecular mechanisms underlying chicken REV infection [23].

This study employed transcriptome sequencing technology to examine alterations in cytokine levels in cell supernatant and lymphocyte gene expression following exposure to goat-derived *P. multocida* type D in PBLs and to explore the genes associated with *P. multocida* type D infection. The findings will enhance our understanding of the alterations in various cytokines that occur after stimulation with *P. multocida* type D and their impact on regulating multiple immune pathways.

## 2. Materials and Methods

### 2.1. Animal Management and Sampling

Three clinically healthy female goats, 6 months old and of similar size, were selected for the study. Serum antibody detection was performed using the Tiger Red Plate Agglutination Kit (Sanofi Kang, Beijing, China) and the goat *Pasteurella multocida* ELISA Kit (RX-AN1100166G, Ruixin, Quanzhou, China). Goats with negative test results were chosen for subsequent experiments. The selected goats were housed individually in enclosures with controlled temperature and humidity. They were provided with a standard diet and allowed free access to food and water.

### 2.2. Bacterial Culture

The HN01 strain, which was isolated early in our laboratory, has been identified as *P. multocida* type D (GenBank accession number: Cp037861.1). The Pm HN01 isolates were reactivated for the current study following the previously outlined procedures [2]. The bacteria were verified through a 16S rRNA PCR. Briefly, the HN01 isolate was recovered from storage at −80 °C and cultured on agar plates containing 5% (*v*/*v*) fresh bovine serum (Hope Bio, Qingdao, China) for 24 h. The strains were identified using PCR with the conserved specific gene *kmt1* (Gene ID: 11963075) of *P. multocida* and the *P. multocida* type D capsule typing gene *CapD*. After confirming the identity of the strain, the remaining individual colonies were inoculated into tryptic soy broth (Sijiqing, Beijing, China) supplemented with 5% (*v*/*v*) newborn bovine serum (Hope Bio, Qingdao, China). When the optical density at 600 nm (OD_600_) reached about 0.7, the plate colony counting method was employed to calculate the number of bacteria and prepare the infection solution. According to previous research, the multiplicity of infection (MOI) was determined to be 10, and the infection duration was set at 12 h [19,24,25].

### 2.3. Isolation of PBLs and Stimulation of PBLs by P. multocida Type D

Three goat anticoagulant blood samples were collected using the jugular vein sampling method as biological replicates. Divide the lymphocytes isolated from each goat into three control groups and three experimental groups. Peripheral blood lymphocytes (PBLs) were isolated following the instructions provided with the goat peripheral blood lymphocyte isolation kit (RX-AN1100166G, Solarbio, Beijing, China). Specifically, the anticoagulant-treated whole blood was mixed with an equal volume of red blood cell diluent. An equal volume of separation liquid was then added to a centrifuge tube, and the diluted blood was layered above the surface of the separation liquid. After centrifugation, the peripheral blood lymphocyte layer was collected into a separate centrifuge tube and washed twice with a detergent solution. The isolated cells were suspended in 1 mL of RPMI 1640 medium (pyg0066, Boster, Shanghai, China), which was enriched with 10% fetal bovine serum (FBS) (164210, Procell, Wuhan, China). Trypan blue staining (C0040-50, Solarbio, Beijing, China) was performed using a blood cell counter with 20 μL of the cell suspension. Giemsa’s stain (L10015105, Yulu, Beijing, China) was employed for the morphological confirmation of PBLs in goat. The cells were seeded into 6-well plates at a concentration of 3 × 10^6^ cells for each well. The experimental group received 100 μL of infection solution, while the control group was supplemented with 100 μL of cell culture medium. Both groups of cells were incubated at 37 °C in a 5% CO_2_ atmosphere for a duration of 12 h.

### 2.4. Determination of Cytokine Levels

Based on previous research, we select five cytokines for ELISA detection [18,26]. According to the instructions of the ELISA kits (ED-75010, ED-75004, ED-75006, ED-202135, ED-756647, Lunchangshuo Biotechnology, Xiamen, China), the concentrations of the IL-6, IFN-γ, IL-10, CXCL10, and IL-17A were detected using a sandwich ELISA. Measure the absorbance (optical density, OD) at a wavelength of 450 nm using a microplate reader (RT-6100, Rayto, Milwaukee, WI, USA). Generate a standard linear curve using OD values and the corresponding concentrations of standard samples. Determine the unknown concentration of each sample by using the standard curve and extrapolating its OD value based on known standard concentrations. Significance analysis was conducted using a two-tailed paired Student’s *t*-test in GraphPad Prism (version 9.5).

### 2.5. RNA Extraction and Transcriptome Sequencing

Total RNA was isolated from the cells utilizing TRIzol (DP424, TIANGEN, Beijing, China). The RNA’s concentration and purity were evaluated with a NanoDrop 2000 spectrophotometer (Thermo Fisher Scientific, Waltham, MA, USA). Additionally, the integrity of the RNA was analyzed using the RNA Nano 6000 detection kit on the Agilent Bioanalyzer 2100 system (Agilent Technologies, Santa Clara, CA, USA). Following a successful quality inspection of the RNA, subsequent analyses were conducted. Following the instructions provided, the sequencing library was constructed with the Hieff NGS^®^ Ultima Dual-mode mRNA Library Prep Kit (12309ES, Yeasen, Shanghai, China). The library’s quality was assessed utilizing the DNA 1000 assay kit from Agilent Technologies (Beijing, China). Sequencing was conducted using the Illumina sequencing platform. Raw reads were subjected to quality control using fastp (version 0.18.0). The clean read results were aligned with the reference genome (GCF_001704415.2) with HISAT2 (version 2.1.0) and normalized using FPKM. Differential gene expression analysis was conducted using edgeR (version 3.12.1) [27]. A log_2_|Fold Change (FC)| ≥ 2 and an adjusted *p*-value < 0.05 were established as thresholds for identifying differentially expressed mRNAs.

### 2.6. Gene Enrichment Analysis

In order to explore the functions of differentially expressed genes (DEGs), we performed a Gene Ontology (GO) analysis utilizing the GO.db database (version 3.14.0). We conducted this enrichment analysis using the KEGG database (Release 101). Both GO and KEGG analyses employed hypergeometric tests to identify significantly enriched components among the differential genes compared to the background. However, when the change in a single gene is minimal, traditional enrichment analysis may yield few or no significant results. To gain a more comprehensive understanding of the role of functional units, we utilized Gene Set Enrichment Analysis (GSEA) software (version 2.2.4). Gene sets were deemed significant if they met the criteria of *p* < 0.05, a False Discovery Rate (FDR) of less than 0.25, and an absolute Normalized Enrichment Score (|NES|) greater than 1.

### 2.7. Interaction Network Analysis

We leveraged the STRING database to examine the differential gene-protein interaction network. The resulting source files were imported into Cytoscape (version 3.9.1) for visual analysis. The ‘MCODE’ plug-in was utilized to pinpoint the most essential functional modules in the PPI network based on default parameters. Determine hub genes by using the MCC algorithm in the CytoHubba plugin. The 15 genes with the highest scores were chosen as central genes based on their centrality, eccentricity, and radiation.

### 2.8. Quantitative qPCR

Based on the transcriptome data, hub genes including *CD86*, *CCL5*, *CD8A*, *CXCL8*, *CTLA4*, *TNF*, *CD274*, *IL-10*, *IL-6*, *CXCL10*, *IFNG*, and *IL-17A* were chosen for qPCR validation. The primer sequences are provided in Supplementary Table S1. Total RNA was converted into cDNA using FastKing gDNA Dispelling RT SuperMix (KR118, TIANGEN, Beijing, China). The qPCR was performed on the QuantStudio™ 6 Flex (ABI, Los Angeles, CA, USA) with the SYBR Green Pro Taq HS Premixed qPCR Kit (AG11735, Accurate Biology, Changsha, China). The relative expression of the genes was normalized to GAPDH, and the expression levels were calculated using the 2^−∆∆Ct^ method.

## 3. Results

### 3.1. Establishment of an Attack Model: Cell Morphology and PCR Identification

**Figure 1:**
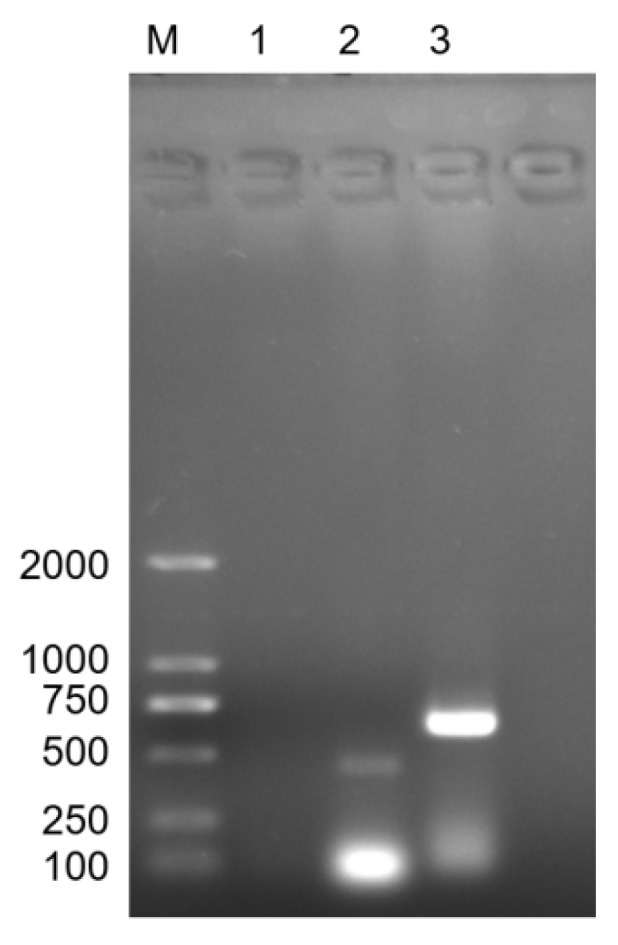
Identification of the PmHN01 strain. M: D2000 DNA marker; 1: blank control; 2: PCR product of the Pm-specific gene *kmt1* primer; 3: PCR product of the HN01-specific primer.

**Figure 2:**
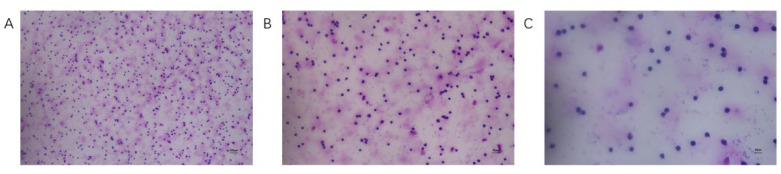
Giemsa’s stain of PBLs. Panels (**A**-**C**) display the staining results of lymphocytes at magnifications of 100×, 200×, and 400×, respectively.

The expected fragment size of *kmt1*, a conserved specific gene of *P. multocida*, in lane 2 is 460 bp, which is located near the 500 bp mark in the electrophoresis image. This finding is generally consistent with the anticipated fragment size. The expected fragment size of *CapD*, a gene associated with the type D capsule of *P. multocida*, in lane 3 is 648 bp, positioned near the 500-750 bp range in the electrophoresis image, also aligning with the expected fragment size. Based on these results, it can be concluded that the PmHN01 strain is *P. multocida* type D (Figure 1). The results of trypan blue staining indicated that the number of PBLs in goats ranged from 1.91 × 10^7^ to 2.5 × 10^7^/mL, with a cell survival rate of 94% (Table S2). Giemsa’s stain revealed that the isolated cells were oval or round in shape, with a rough cell surface, reduced cytoplasm, and a large nucleus that appeared purple-blue or blue. The nuclear chromatin was dense and evenly distributed, consistent with the morphological characteristics of lymphocytes, confirming that we successfully isolated lymphocytes with high purity (Figure 2).

### 3.2. Analysis of Cytokine Detection Using the ELISA Method

**Figure 3:**
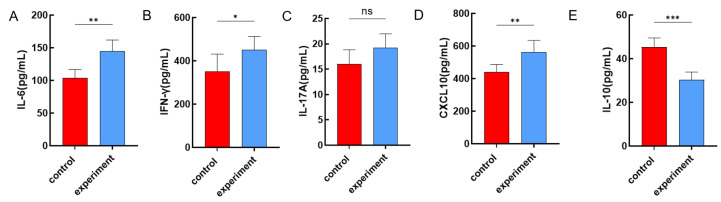
Cytokine level detection results. The vertical axis represents cytokine concentration, with the red bar indicating the control group and the blue bar representing the experimental group. (**A**) IL-6 ELISA Results. (**B**) IFN-γ ELISA Results. (**C**) IL-17A ELISA Results. (**D**) CXCL10 ELISA Results. (**E**) IL-10 ELISA Results. *** *p* < 0.001; ** *p* = 0.001 to < 0.01; * *p* = 0.01 to 0.05; ns = no significant.

We selected pro-inflammatory cytokines IL-6, IFN-γ, IL-17A, CXCL10, and anti-inflammatory cytokine IL-10 [28]. The analysis showed significant differences (*p* < 0.05) in cytokine levels of IL-6, IFN-γ, CXCL10, and IL-10 between the control and experimental groups. IL-6, IFN-γ, and CXCL10 levels were elevated in the experimental group, while IL-10 levels decreased. No significant difference was found in IL-17A levels between the groups (*p* = 0.06) (Figure 3). The detection results of ELISA suggest that within 12 h after infection, the immune response induced by the bacterium in PBLs is mainly through the pro-inflammatory pathway.

### 3.3. Evaluate the Quality of Sequencing

The sequencing data, detailed in Supplementary Table S3, comprised six samples (three experimental groups and three control groups) and generated 242 million raw reads. Following the removal of low-quality reads and joint sequences, 241 million clean reads remained. In each sample, 95.73% to 96.07% of the reads were successfully mapped, with 92.01% to 92.52% mapped to unique reads, and 3.31% to 4.13% mapped to multiple reads. The Q30 metric, which indicates a sequencing error rate of 0.1%, was above 95.37% in this study. This high Q30 value demonstrates that the quality of the sequencing data is excellent, and the samples are suitable for the objectives of this research.

### 3.4. Identification of DEG

**Figure 4:**
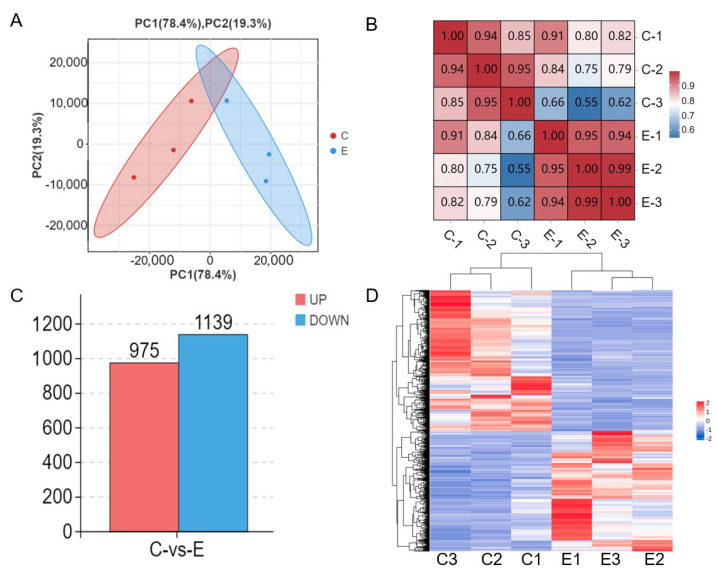
Differential expression of DEGs. (**A**) PCA of DEG transcripts. X−axis: PC1 coordinates denote the first principal component. Y−axis: PC2 coordinates represent the second principal component. The red and blue dots in the figure denote the control group and experimental group, respectively. (**B**) Heatmaps illustrating the relationships among different samples. The horizontal and vertical axes represent each sample. A closer proximity to red signifies a stronger correlation, whereas a closer proximity to blue indicates a weaker correlation. (**C**) In the bar chart, blue shows downregulated DEGs and red shows upregulated DEGs. (**D**) In the differential gene clustering heatmap, each column corresponds to a sample, and each row corresponds to a gene. Red signifies increased gene expression, while blue represents decreased expression levels.

We carried out principal component analysis (PCA) and correlation analysis on six sequencing samples, comprising three experimental groups and three control groups, at the transcriptome level. The PCA revealed distinct clustering patterns for the experimental and control groups (Figure 4A). The sample correlation analysis indicated strong repeatability among the repeated samples within each group (Figure 4B). We utilized edgeR software to analyze the DEGs between the experimental and control groups, resulting in 2114 DEGs. Of these, 975 were upregulated, while 1139 were downregulated. A bar chart was employed to visualize these DEGs (Figure 4C). Additionally, we performed hierarchical clustering of the differential gene expression patterns and presented the clustering results using heat maps (Figure 4D). The hierarchical clustering analysis demonstrated notable variations in the expression patterns of differentially expressed genes between the experimental and control groups. This suggests that the stimulation of *P. multocida* type D is linked to notable changes in the gene expression profile of PBLs compared to the control samples.

### 3.5. GO, KEGG, and GSEA Analysis of DEGs

**Figure 5:**
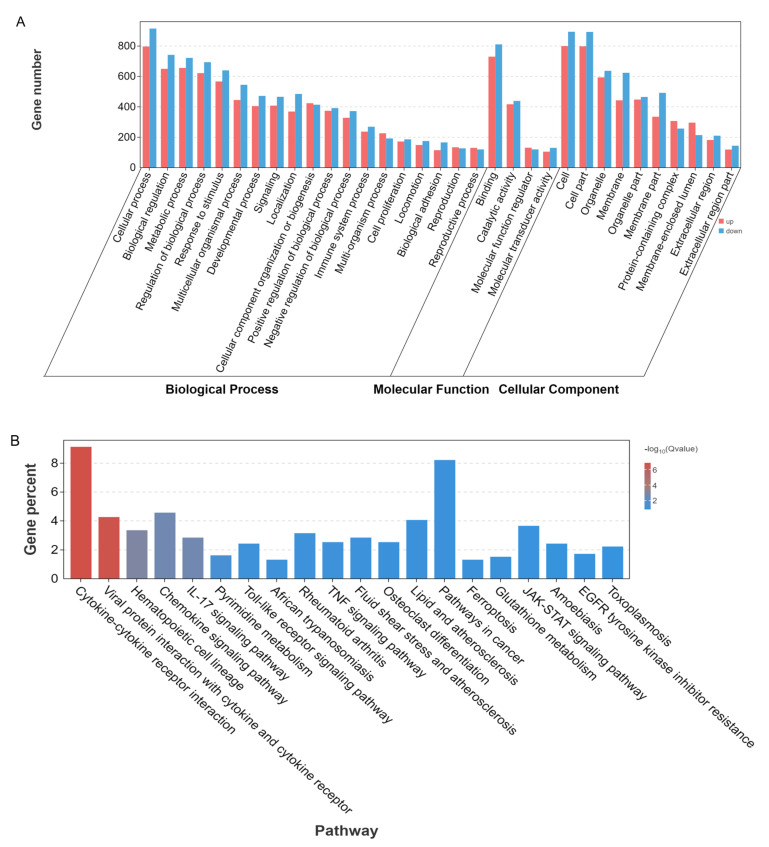
GO and KEGG annotation. (**A**) GO enrichment classification bar chart: X-axis: secondary GO terms; Y-axis: number of differentially expressed genes associated with each term. Red: upregulated genes; blue: downregulated genes. (**B**) KEGG enrichment bar chart: X-axis: pathways; Y-axis: percentage of DEGs in the pathway compared to the total DEGs. The color of the column represents the degree of enrichment significance of the pathway.

To gain deeper insights into the function of DEGs in the process of PBLs in *P. multocida* type D, we performed GO and KEGG analyses. The GO analysis showed that the DEGs were primarily enriched in the biological process category. The biological processes predominantly involved cellular processes, biological regulation, and metabolic processes. In terms of molecular function, the DEGs are mainly related to binding and catalytic activity, while the classification of cellular components primarily encompassed cells, cell parts, and organelles (Figure 5A). The KEGG enrichment analysis indicated that the DEGs were significantly associated with pathways such as cytokine-cytokine receptor interaction, viral protein-cytokine, chemokine signaling pathway, and IL-17 signaling pathway, among others (Figure 5B).

The GO enrichment analysis of upregulated DEGs revealed that the biological processes that were significantly enriched primarily included cytokine-mediated signaling pathways and cellular responses to cytokine stimuli (Figure S1A). The KEGG pathway annotation results identified several important pathways, including cytokine-ccytokine receptor interactions, viral protein-cytokine interactions, and the IL-17 signaling pathway (Figure S1B). GO enrichment analysis of downregulated DEGs showed that the enriched biological processes were mainly related to cell adhesion and biological adhesion (Figure S2A). The KEGG pathway annotation results indicate that the important pathways for DEG enrichment include lysosomal function and glutathione metabolism (Figure S2B).

**Figure 6:**
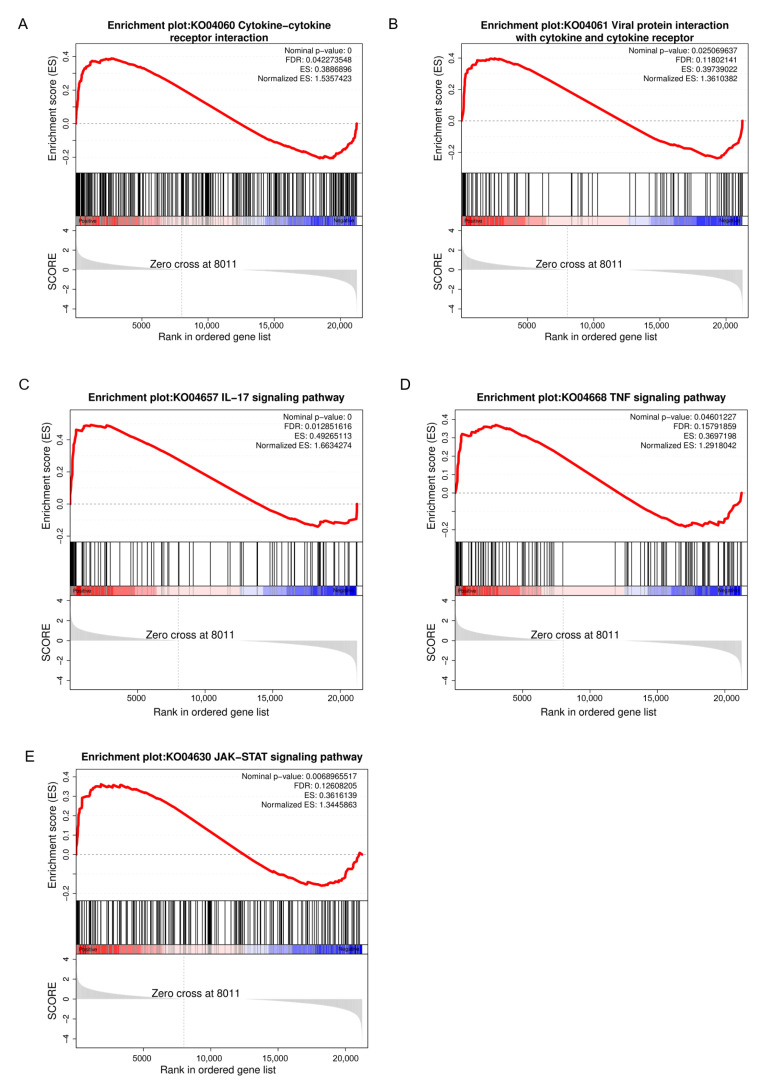
GSEA analysis of key pathways. Top figure: Curve illustrating the increases and decreases in the accumulation process of ES values. Middle figure: Positions of target gene set members within the ranking of all genes, indicated by black vertical lines. Red bars: Genes that are positively correlated with the experimental group. Blue bars: Genes that are negatively correlated with the control group. Bottom figure: Actual values of the ranking indicators for genes, arranged from highest to lowest. (**A**) IL-17 Signaling Pathway. (**B**) Cytokine-cytokine receptor interactions. (**C**) Signaling pathway of the interaction between viral proteins and cytokines and cytokine receptors. (**D**) JAK-STAT signaling pathway. (**E**) TNF signaling pathway.

Our analysis showed that IL-17 signaling, cytokine-cytokine receptor interactions, viral protein interactions with cytokines and their receptors, as well as the JAK-STAT and TNF signaling pathways, are closely related to immune response and significantly enriched in both KEGG and GSEA (Figure 6).

### 3.6. PPI Network Analysis of DEGs

**Figure 7:**
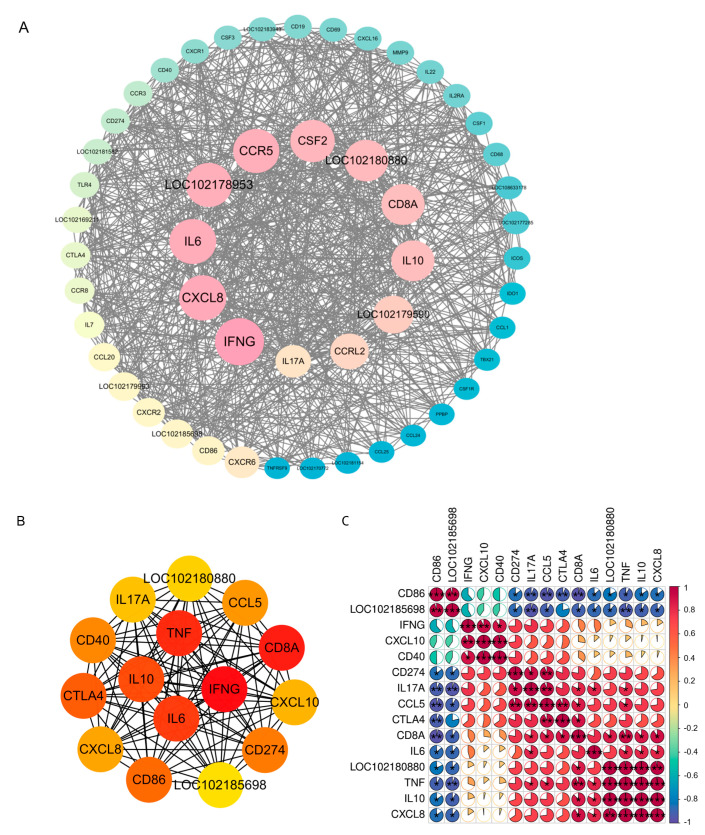
Important modules and hub genes. (**A**) The most important modules are evaluated using betweenness centrality. The circles transition from pink to yellow to blue according to the ranking from high to low. The larger the circles and the font, the higher the ranking. (**B**) The top 15 hub DEGs identified using the MCC algorithm. In the image, genes are colored according to their scores, transitioning from red to yellow as the scores decrease. (**C**) Correlation analysis of the 15 hub DEGs. where red indicates a positive correlation and blue indicates a negative correlation. The larger the sector, the stronger the correlation it represents. *** indicates *p* < 0.001; ** indicates *p* < 0.01; * indicates *p* < 0.05.

To evaluate the interaction between differentially expressed genes at the protein level, we utilized the STRING online platform to construct a protein-protein interaction network. To identify key modules that could have significant roles within the network, we loaded the constructed PPI network into Cytoscape software. Using the MCODE plugin, we analyzed the key functional modules and identified 51 differentially expressed genes in the most significantly enriched module. We further assessed the importance of these 51 genes using the betweenness centrality metric in the CytoNCA plugin (Figure 7A). Afterward, we performed KEGG and GO analyses on these genes. The GO analysis indicated that the genes were primarily linked to cytokine-mediated signaling pathways, immune responses, and chemokine-mediated signaling pathways (Figure S3A). The KEGG pathway analysis indicated that they were primarily engaged in cytokine–cytokine receptor interactions, interactions between viral proteins, cytokines, and cytokine receptors, the chemokine signaling pathway, and the IL-17 signaling pathway (Figure S3B).

The MCC algorithm, utilizing the CytoHubba plugin, was employed to identify the top 15 central genes (Figure 7B). We observed that several key genes exhibited a strong correlation with the characteristic target genes of the IL-17 signaling pathway. A correlation coefficient analysis was performed on the central genes (Figure 7C). The Pearson correlation analysis revealed that *IL-17A* has an antagonistic effect on *CD86* and *LOC102185698* (with coefficients less than 0.96), while it demonstrates a synergistic effect on *CCL5*, *CD274*, *CD8A*, *IL-6*, and *TNF* (with coefficients greater than 0.81).

### 3.7. Validation of RNA-Seq

**Figure 8:**
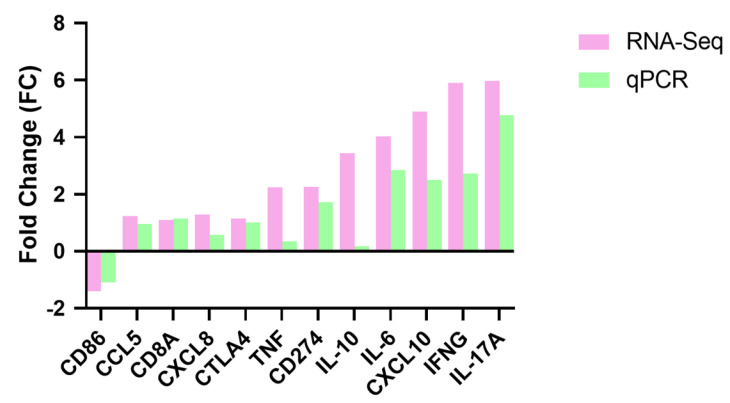
qPCR validation of DEGs. The green bars represent qPCR results, while the pink bars indicate RNA sequencing results.

To confirm the reliability of our RNA-seq analysis, we chose hub genes (*CD86*, *CCL5*, *CD8A*, *CXCL8*, *CTLA4*, *TNF*, *CD274*, *IL-10*, *IL-6*, *CXCL10*, *IFNG*, and *IL-17A*) for further examination (Figure 8). The results indicated that the qPCR validation was aligned with the RNA-seq findings, thereby affirming the accuracy and dependability of the RNA-seq results.

## 4. Discussion

The lipopolysaccharide (LPS) of *P. multocida* plays a crucial role in stimulating the host’s immune response [29]. The lipid A component of LPS is recognized by Toll-like receptors (TLRs), which activate intracellular signaling cascades and the NF-κB signaling pathway. This activation leads to the production of various pro-inflammatory cytokines, notably IL-6 and TNF-α [30]. IL-6, a significant pro-inflammatory factor, is produced by T lymphocytes, monocytes/macrophages, and vascular endothelial cells. It not only stimulates T cell proliferation and activates cytotoxic T cells but also enhances the proliferation of activated B cells and promotes antibody secretion [31]. During bacterial infections, IL-6 is vital for activating the STAT signaling pathway, enhancing neutrophil recruitment, and reducing the bacterial load, thereby playing a central role in the host’s defense against *P. multocida* [32].

CD4^+^ and CD8^+^ T cells are key players in the immune response to *P. multocida*. These cells secrete IFN-γ through receptor-dependent and cytokine-dependent mechanisms. The interaction between the T cell receptor (TCR) and its corresponding antigen triggers the activation of several Src-family tyrosine kinases, ultimately leading to the activation of mitogen-activated protein kinases (MAPK). This activation stimulates the transcription factors Jun and Fos, enhancing the transcriptional expression of IFN-γ [33]. Notably, studies have shown that a combination of IL-12 and IL-18 can induce IFN-γ secretion in Th1-differentiated CD4^+^ T cells in vitro, even in the absence of antigen or antibody-mediated TCR activation. This indicates that cytokine-mediated T cell activation can occur independently of TCR engagement [34]. The majority of IFN-γ signaling pathways are mediated through the JAK-STAT pathway [35]. The SOCS family of proteins plays a role in inhibiting this pathway [36]. Our data further indicate that the JAK-STAT signaling pathway is particularly active in our observations, highlighting its importance in the immune response to *P. multocida*.

Similarly, T cell mitogens were used to activate PBMCs from Saanen goats, followed by infection with PPRV strains of varying virulence. Transcriptomic analysis revealed changes in the IL-17 signaling pathway during the infection period [21]. In another study, transcriptome sequencing of peripheral blood lymphocytes from dairy cows infected with *Bovine viral diarrhea virus* (BVDV) for 12 h showed significant enrichment of pathways such as cytokine-cytokine receptor interaction, complement and coagulation cascades, and IL-17 signaling. PPI analysis highlighted the critical roles of *IFN-γ*, *STAT1*, *CXCL10*, and *IL17A* [37]. Furthermore, high-throughput RNA sequencing was performed on human PBMCs stimulated with Mtb-Ag for 6 h, which identified the enrichment of signaling pathways including TNF, IL-17, and JAK-STAT. PPI analysis indicated that genes such as *IL10*, *IL6*, and *CXCL8* played crucial roles in these interactions [20]. These studies consistently demonstrate that the IL-17 signaling pathway plays a significant role in infections caused by various pathogens.

IL-17A is essential for providing protection in the host’s defense against *P. multocida* infection. It synergistically activates macrophages in conjunction with IFN-γ, promoting the expression of inducible nitric oxide synthase (iNOS) and the release of nitric oxide (NO), which contributes to bacterial clearance [38]. IL-17A binds to its receptor IL-17RA, forming a receptor complex [39]. This complex recruits E3 ubiquitin ligase and activates the TNF receptor-associated factor (TRAF) family [40]. This complex activates multiple downstream pathways, driving the transcription of target genes encoding matrix metalloproteinases, antimicrobial peptides, pro-inflammatory cytokines, and chemokines [41].

Chemokines play a critical role in the host’s response to *P. multocida* infection by recruiting and activating leukocytes at the site of infection. IFN-γ, produced by Th1 cells, triggers the synthesis of CXCL10 across different cell types. CXCL10 attracts monocytes and activated T lymphocytes to the site of inflammation, promoting a selective enhancement of the Th1 response and increasing IFN-γ gene expression. This creates a positive feedback loop between Th1 cells and resident cells that produce CXCL10 [42]. CXCL10 is a multifunctional molecule that promotes the chemotactic activity of CXCR3-expressing cells, contributing to the recruitment of immune cells [43]. Previous transcriptome analyses of lungs infected with *P. multocida* in mice and goats have demonstrated the activation of chemokine pathways [2,44]. Notably, *CCL25* and *CCL24* expression was downregulated, while *CCL20*, *CCL1*, *CCL5*, *CXCL8*, *CXCL16*, and *CXCL10* were upregulated. CXC chemokines are chiefly involved in attracting neutrophils, whereas CC chemokines play a primary role in recruiting monocytes and different lymphocyte subsets [45]. These chemokines are essential for orchestrating the immune response to *P. multocida* infection, facilitating the recruitment and activation of immune cells at the infection site.

## 5. Conclusions

In summary, we characterized the immune response of goat PBLs infected with goat-derived *P. multocida* type D for 12 h. We utilized ELISA, RNA sequencing, and qPCR technologies to investigate the upregulation of cytokines IL-6, IFN-γ, CXCL10, and IL-17A at both the gene and protein levels. We identified the key pathways and genes associated with the response of PBLs to this infection, thereby determining the consistency of our research results at both the genetic and protein levels. This study provides deeper insights into the immune responses induced by *P. multocida* in the PBLs of goats, which will facilitate the development of specific diagnostic markers and vaccine adjuvants aimed at immune activation.

## Data Availability

The raw sequence data reported in this paper have been deposited in the Genome Sequence Archive in the National Genomics Data Center, China National Center for Bioinformation/Beijing Institute of Genomics, Chinese Academy of Sciences, under accession number CRA019167, which are publicly accessible at https://bigd.big.ac.cn/gsa (accessed on 25 September 2024).

## Supplementary Materials

The following supporting information can be downloaded at: https://www.mdpi.com/article/10.3390/microorganisms12122618/s1, Figure S1: GO and KEGG analysis of upregulated. Figure S2: GO and KEGG analysis of downregulated genes. Figure S3: GO and KEGG analysis of genes in important modules. Table S1: Primers for qPCR; Table S2: Cell survival rate statistics by trypan blue staining; Table S3: Reliability of sequencing data.
