# Supplementary material for: Pasteurella multocida Serotype D Infection Induces Activation of the IL-17 Signaling Pathway in Goat Lymphocytes
Source: Microorganisms. 2024 Dec 18;12(12):2618. doi: 10.3390/microorganisms12122618 (PMC11678448; doi:10.3390/microorganisms12122618)
Supplement: Supplementary file 1 [file microorganisms-12-02618-s001.zip › microorganisms-3341242-supplementary.pdf]

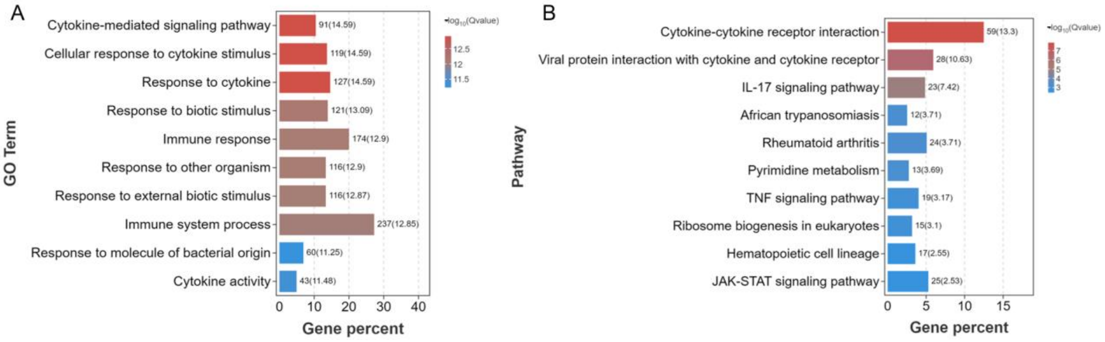

Figure S1. GO and KEGG Analysis of Up-regulated. (A) X-axis: The percentage of target genes in the GO term to the total number of genes in the target gene set. Y-axis: GO term name. The color of the column represents the degree of enrichment significance of the GO term. (B) X-axis: The percentage of target genes in the total target genes in the pathway. Y-axis: Path name. The color of the column represents the degree of enrichment significance of the pathway.

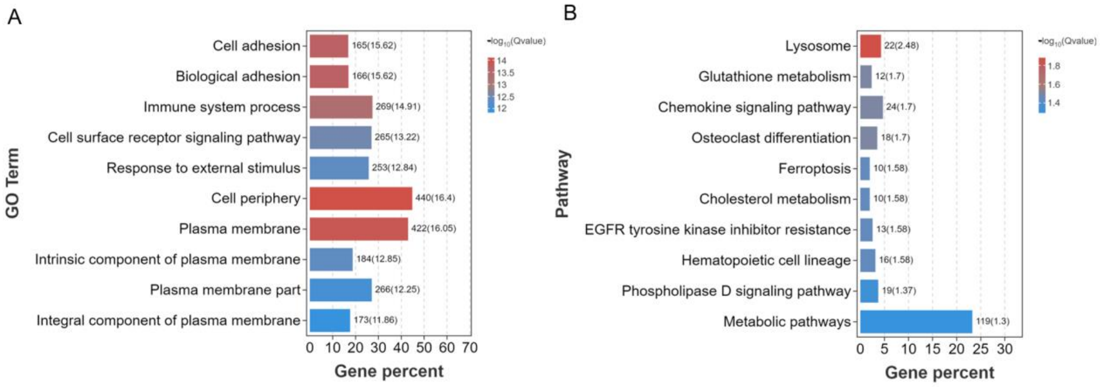

Figure S2. GO and KEGG Analysis of Down-regulated Genes. (A) X-axis: The percentage of target genes in the GO term to the total number of genes in the target gene set. Y-axis: GO term name. The color of the column represents the degree of enrichment significance of the GO term. (B) X-axis: The percentage of target genes in the total target genes in the pathway. Y-axis: Path name. The color of the column represents the degree of enrichment significance of the pathway.

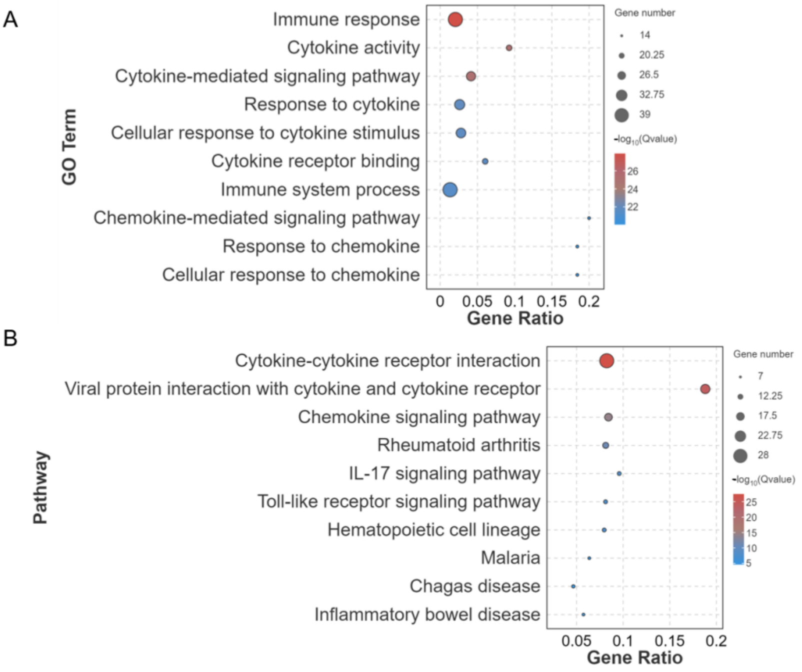

Figure S3. GO and KEGG Analysis of Genes in Important Modules. (A) GO Enrichment Bubble Plot: The vertical axis represents the GO term, while the horizontal axis indicates the enrichment factor. The size of the bubbles corresponds to

the quantity of genes, and a deeper red color signifies a smaller Q value. (B) KEGG Enrichment Bubble Chart: The vertical axis denotes the pathway, and the horizontal axis reflects the enrichment factor. The size of the bubbles again represents quantity, with a deeper red color indicating a smaller Q value.

Table S1 Primers for qPCR

Gene name	Forward primer (5`-3`)	Reverse primer (5`-3`)
CD8A	GCCGACGCAGAAGGAGACC	TGGGACGAGAGATACATTAGGAAGG
IL10	TGAAAGGAGAAGGGATAGGGAAGAG	TGGCTGGAGACTCACACTTGG
CXCL8	GGCCACACTGCGAAAATTCA	CTTGCTTCTCAGCTCTCTTCA
IL6	TCGCAGGTCTAATAACCACTCCAG	CAAGGCTTCTCAGGATGATGATAACC
CXCL10	AACACGGAAAGAGGCATAATCACTG	AGAACTGAAGTCCATGAACAATTAGGG
CCL5	ATCACCAGGAAGAACCGTCAGG	TAGGACAAGAGCGAGAAGCAGAG
CD274	CTCAGCCTGTGGTTTGGGTTTATC	TCCTTCTTCCTCTGTCCTCTTCTTTC
CD86	AGACAGACAGACGGACAGACAAG	AAGGAACGGTGGAAACAGAAAGC
IL17A	GAACTGCATCTATGTCACTGCT	CAGGTTGACCCTCACATGCT
GAPDH	CCTGGAGAAACCTGCCAAGTA	AAGGTAGAAGAGTGAGTGTCGC
CTLA4	AGGCTGGCTCTGTCACATCTGG	CCTGCGTCGTAATGCTGCTTCC

IFNG	AAGTTCTTGAACGGCAGCTCTGAG	TTGGCGACAGGTCATTCATCACC
TNF	CAACGGCGTGGAGCTGAAAG	TGAAGAGGACCTGCGAGTAGATG

Table S2: Cell survival rate statistics by trypan blue staining

Experimental Goat Number	Total lymphocyte count	Cell survival rate
A	2.5×10^{7}	0.95
B	1.91×10^{7}	0.94
C	2.2×10^{7}	0.95

Table S3 Reliability of sequencing data

Sample	RawDatas	CleanData(%)	AF_Q30(%)	Mapped_Reads(%)	Unique_Mapped(%)	Multiple_Mapped(%)
C-1	35982000	35754394 (99.37%)	5066639008 (95.88%)	53502 (0.15%)	32873725 (92.08%)	1303989 (3.65%)
C-2	36138238	35935708 (99.44%)	5097949743 (95.90%)	56934 (0.16%)	33193471 (92.52%)	1254227 (3.50%)
C-3	46603694	46377758 (99.52%)	6581059394 (95.77%)	102900 (0.22%)	42794447 (92.48%)	1533113 (3.31%)
E-1	37784638	37633324 (99.60%)	5385637547 (96.58%)	49466 (0.13%)	34664662 (92.23%)	1440445 (3.83%)
E-2	48365184	48182618 (99.62%)	6842548989 (95.63%)	91726 (0.19%)	44128819 (91.76%)	1986104 (4.13%)
E-3	38083270	37943104 (99.63%)	5371761047 (95.50%)	101566 (0.27%)	34818961 (92.01%)	1537058 (4.06%)
